# Diffuse Idiopathic Skeletal Hyperostosis of Upper Cervical Vertebrae: A Case Report of a Rare Anatomical Presentation During Bone Retrieval

**DOI:** 10.7759/cureus.94022

**Published:** 2025-10-07

**Authors:** Balaaji Thirumaran, Medora Dsouza Dias

**Affiliations:** 1 Anatomy, Goa Medical College and Hospital, Panaji, IND

**Keywords:** alar ligament, anterior longitudinal ligament, diffuse idiopathic skeletal hyperostosis, forestier’s disease, osteophytes

## Abstract

Diffuse idiopathic skeletal hyperostosis (DISH), also known as Forestier’s disease, is a systemic skeletal disorder characterized by ossification of spinal and extraspinal ligaments. While DISH commonly affects the thoracic and lumbar spine, upper cervical involvement (C1-C3) is rare. During routine dissection and subsequent bone retrieval for the osteology library at Goa Medical College, ossification of the anterior longitudinal ligament (ALL) and the alar ligament was observed in the upper cervical vertebrae. Upper cervical DISH may remain asymptomatic or manifest as dysphagia, airway obstruction, and restricted neck movement. Radiological features include the characteristic “dripping candle wax” appearance. Diagnosis is based on Resnick’s criteria. Although often incidental, upper cervical DISH has significant clinical implications due to its proximity to vital structures such as the esophagus and trachea. Awareness of this rare presentation is crucial for timely diagnosis and management.

## Introduction

Diffuse idiopathic skeletal hyperostosis (DISH), or Forestier’s disease, is a non-inflammatory systemic skeletal disorder characterized by ossification of spinal and extraspinal ligaments and entheses. First described by Forestier and Rotes-Querol in 1950, DISH typically involves the thoracic spine, followed by the cervical region than the lumbar region. The upper cervical spine (C1-C3) is rarely affected, making its recognition important due to the proximity to critical structures such as the esophagus, trachea, and neurovascular bundles [1].

The condition is associated with older adults, typically above 50 years of age, male sex, obesity, diabetes mellitus, and metabolic syndrome [2]. While DISH is often asymptomatic, it can present with stiffness, dysphagia, and, in severe cases, airway compromise [3]. The diagnosis is established radiologically, often using Resnick’s criteria [4]. This report presents a rare anatomical variation of DISH involving C1-C3, discovered incidentally during bone retrieval for the osteology library at Goa Medical College, Goa, India.

## Case presentation

During routine dissection and subsequent bone retrieval for osteology collection at the Department of Anatomy, Goa Medical College, Bambolim, Goa (GMCIEC/2024/233), we encountered a C1-C3 cervical vertebral specimen demonstrating the following features: ossified anterior longitudinal ligament (ALL) along the anterior surface of the vertebral bodies, ossified alar ligament forming prominent osteophytes, and continuous ossification, creating bony bridges across adjacent vertebrae. This finding was incidental, and no clinical history was available as the specimen belonged to a cadaver prepared for academic purposes.

Imaging findings

**Figure 1:**
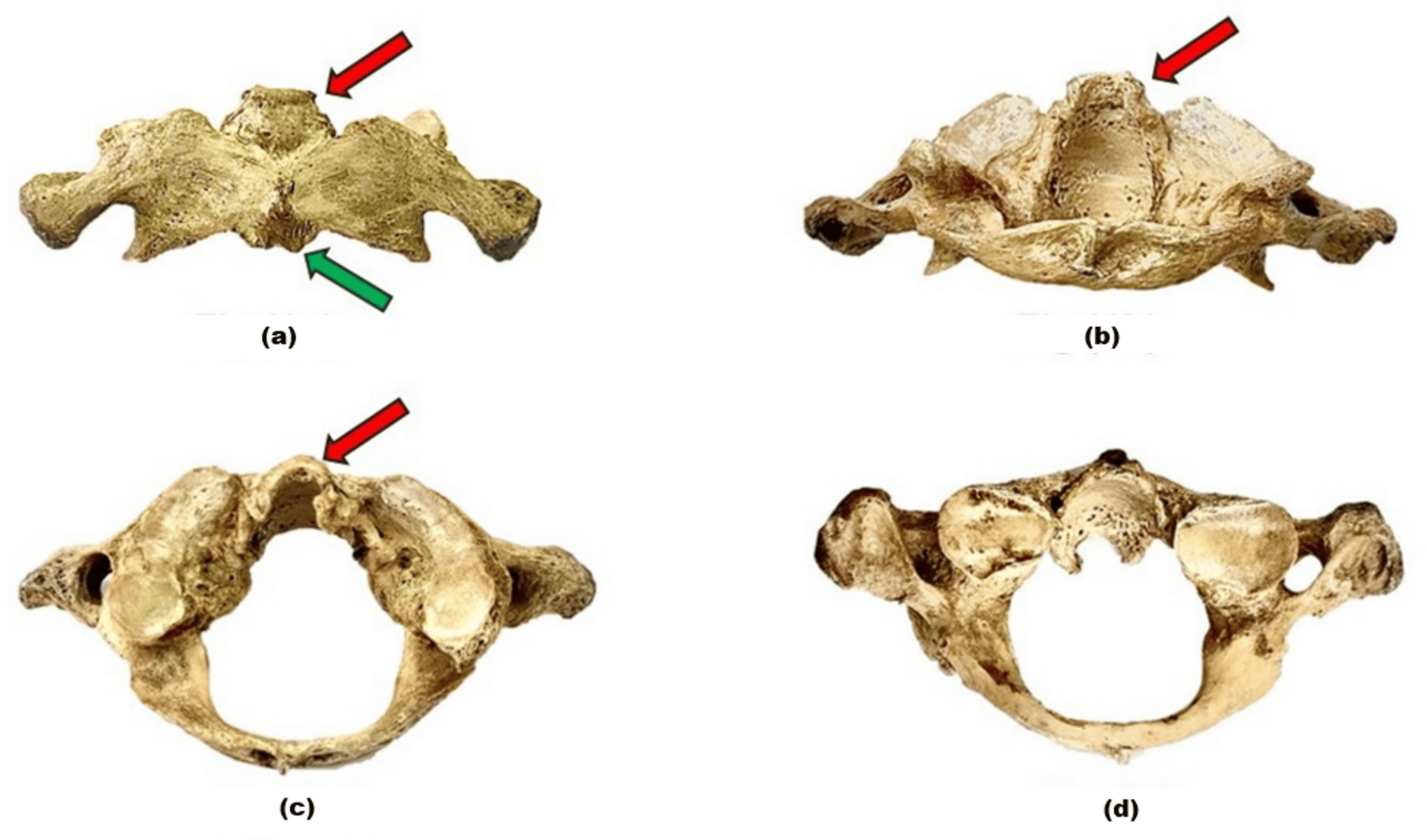
The atlas (C1 vertebra) viewed from different aspects. (a) shows the anterior view; osteophyte of the anterior longitudinal ligament in the anterior arch is identified (green arrow) along with osteophyte of the alar ligament (red arrow). (b) demonstrates the posterior view of osteophyte of the alar ligament (red arrow). (c) shows the superior view of the C1. (d) shows the inferior view.

**Figure 2:**
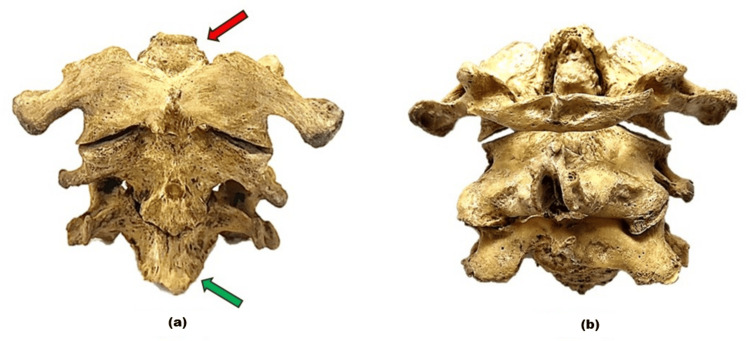
Upper cervical vertebrae (C1-C3) in different views. (a) shows the anterior view of the first three cervical vertebrae (C1-C3). The atlas (C1) with its osteophyte of the anterior longitudinal ligament (green arrow) and osteophyte of the alar ligament (red arrow). (b) presents the posterior view of the cervical vertebrae (C1-C3).

**Figure 3:**
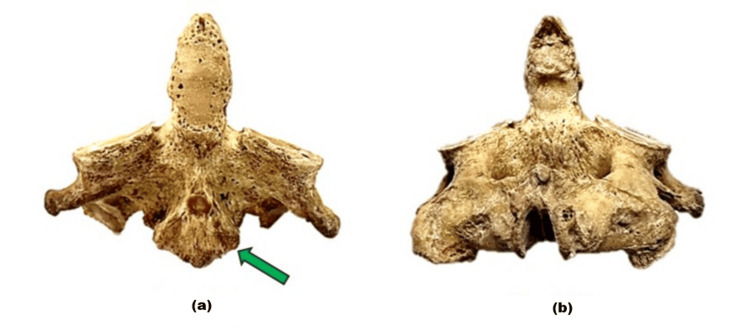
The axis (C2 vertebra) viewed from different aspects. (a) shows the anterior view of the axis (C2 vertebra), where the prominent osteophyte of the anterior longitudinal ligament (green arrow). (b) presents the posterior view of the axis.

**Figure 4:**
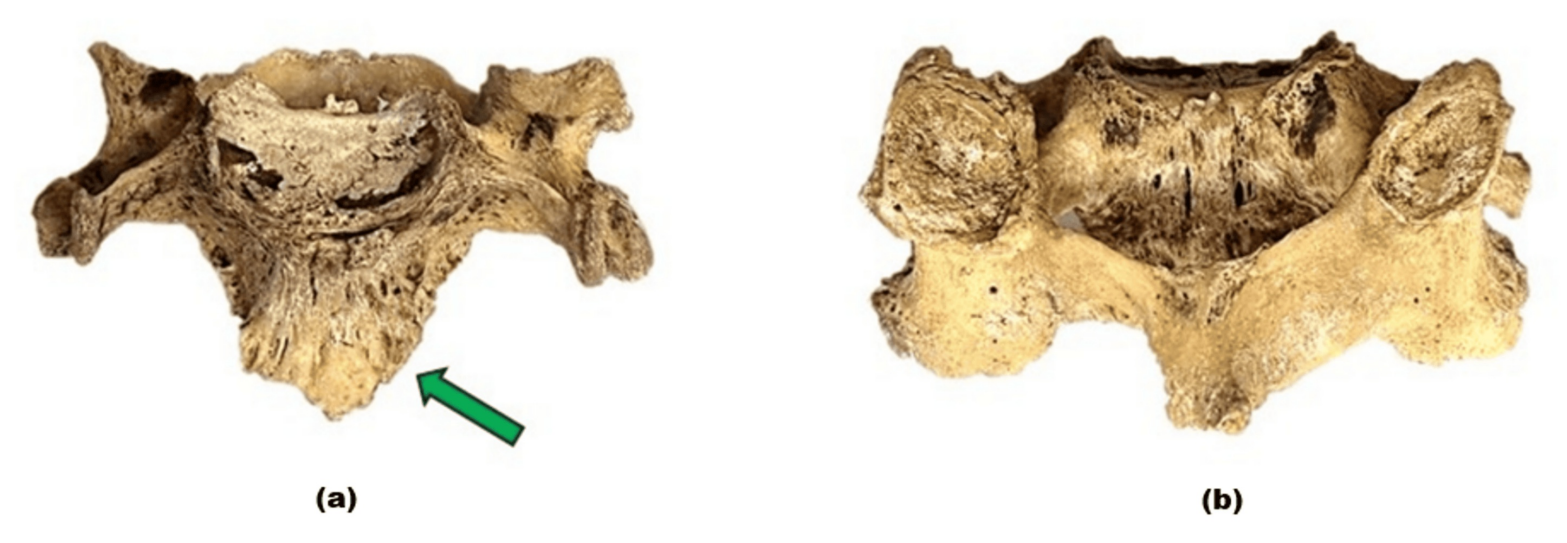
The C3 vertebra viewed from different aspects. (a) shows the anterior view of the C3 vertebra, where the prominent osteophyte of the anterior longitudinal ligament (green arrow). (b) presents the posterior view of the axis.

In the atlas (C1) (Figure 1), osteophytes of the ALL (green arrow) and alar ligament (red arrow) are demonstrated in anterior and posterior views. In the upper cervical vertebrae (C1-C3) (Figure 2), similar changes are noted with clearer visualization of C1 osteophytes. The axis (C2) (Figure 3) shows a prominent osteophyte of the ALL (green arrow), while the C3 vertebra (Figure 4) also demonstrates an ALL osteophyte (green arrow).

## Discussion

DISH is a systemic, non-inflammatory condition characterized by calcification and ossification of ligaments and entheses, most prominently the ALL. The disease most frequently involves the middle to lower thoracic spine, but cervical spine involvement is also documented in approximately 6%-12% of cases [5]. Within the cervical region, upper cervical spine (C1-C3) disease is exceptionally rare, reported in only 2%-4% of cases [2]. The clinical relevance of upper cervical involvement lies in its potential to cause compressive symptoms due to the close anatomical relationship of osteophytes to the pharynx, esophagus, and trachea [3].

From a clinical perspective, patients with cervical DISH may remain asymptomatic for prolonged periods, but progressive ossification can lead to significant morbidity. Dysphagia is one of the most common clinical manifestations, arising from mechanical compression of the esophagus by anterior cervical osteophytes. In more advanced cases, patients may also develop airway obstruction when osteophytes encroach upon the trachea, resulting in stridor or respiratory distress. Another important consequence is restriction of cervical mobility, which results from bridging ossifications and may significantly impair daily activities and quality of life. Rarely, neurological symptoms may develop if associated degenerative changes cause spinal cord or nerve root compression [6].

The diagnosis of DISH is established using Resnick’s criteria, which remain the gold standard. These include (1) ossification of the ALL in at least four contiguous vertebrae, (2) preservation of the intervertebral space, (3) preservation of the facet joint, and (4) absence of sacroiliac joint involvement [4]. Radiological imaging typically demonstrates the pathognomonic “dripping candle wax” appearance of ossification along the anterior aspect of vertebral bodies. Preservation of disc spaces, along with the absence of sacroiliac involvement, reinforces the distinction from inflammatory spondyloarthropathies [4].

Management of cervical DISH is individualized depending on the severity of symptoms. Most patients benefit from conservative measures such as analgesics, non-steroidal anti-inflammatory drugs, and physiotherapy aimed at improving neck mobility and alleviating pain. However, conservative treatment may be inadequate in patients with severe dysphagia, recurrent aspiration, or airway compromise [7]. In such cases, surgical resection of anterior osteophytes is indicated and has been shown to provide significant symptomatic relief. Postoperatively, recurrence of osteophyte formation is possible but usually progresses slowly, often without recurrence of compressive symptoms [8].

Thus, while DISH is generally considered a benign condition, cervical and especially upper cervical involvement may have serious clinical implications due to the risk of dysphagia and airway obstruction. Imaging studies, including CT and MRI, are valuable in diagnosing DISH in this region, as they provide detailed visualization of ossification around C1-C2 and potential encroachment on surrounding structures [9]. Early recognition of this rare manifestation, careful differentiation from other spinal disorders, and timely intervention are essential for preventing potentially life-threatening complications [8].

## Conclusions

This case highlights a rare anatomical variation of DISH involving the upper cervical vertebrae (C1-C3). Though often incidental, such findings are clinically significant because of the potential for esophageal and tracheal compression. Awareness of this condition aids in accurate diagnosis, differentiation from other spondyloarthropathies, and appropriate management to prevent complications.
